# Soy isoflavones induces mitophagy to inhibit the progression of osteosarcoma by blocking the AKT/mTOR signaling pathway

**DOI:** 10.1186/s10020-024-00778-y

**Published:** 2024-01-08

**Authors:** Ziang Zheng, Xinghan Zhao, Bo Yuan, Shan Jiang, Rushan Yan, Xiaowei Dong, Qijun Yao, Haidong Liang

**Affiliations:** Department of Bone and Soft Tissue Repair and Reconstructive Surgery, The Second Hospital of Dalian Medical University, No. 467 Zhongshan Road, Dalian, 116000 Liaoning China

**Keywords:** Osteosarcoma, Soy isoflavones, Mitophagy, Autophagy, AKT/mTOR pathway

## Abstract

**Background:**

Soy isoflavones (SI) is a natural bioactive substance exhibiting beneficial effects on human health. This study aims to elucidate the therapeutic potential of SI in the treatment of osteosarcoma (OS) and to investigate the underlying mechanisms, particularly focusing on mitophagy.

**Methods:**

The effects of SI on the proliferation, apoptosis, migration, and invasion of U2OS cells were analyzed. Mitophagy was assessed through multiple parameters: mitochondrial autophagosomes, mitochondrial membrane potential, autophagy-related proteins, reactive oxygen species (ROS), and oxygen consumption rate (OCR). Protein levels related to apoptosis, autophagy, and the AKT/mTOR pathway were analyzed using western blot. The therapeutic efficacy of SI was further identified using a mouse tumor xenograft model. Cell apoptosis and proliferation in tumor xenografts were detected by TUNEL staining and immunohistochemistry (IHC), respectively.

**Results:**

SI dose-dependently suppressed the viability, colony formation, migration, and invasion of U2OS cells, and enhanced the apoptosis. SI also dose-dependently induced mitophagy in OS cells, evidenced by an increase in autophagosomes and ROS levels, a decrease in mitochondrial membrane potential and OCR, and concomitant changes in autophagy-related proteins. Mdivi-1, an inhibitor of mitophagy, reversed the anti-tumor effects of SI on U2OS cells. In addition, SI blocked the AKT/mTOR pathway in U2OS cells. SC-79, an AKT agonist, reversed the effect of SI on inducing mitophagy. Moreover, SI also promoted cell apoptosis and mitophagy in tumor xenografts in vivo*.*

**Conclusions:**

SI induces mitophagy in OS cells by blocking the AKT/mTOR pathway, contributing to the inhibition of OS.

## Background

Osteosarcoma (OS) is a frequent primary bone tumor, characterized by the formation of osteoid or immature bone tissues (Yang et al. 2022). Because OS usually occurs in growing bones, it is more frequent in children and adolescents (Shabani et al. 2019). A second peak in the incidence of OS is observed in individuals around the age of 60 years (Beird et al. 2022). Clinically, surgical intervention coupled with neoadjuvant and postoperative chemotherapy is the standard treatment for OS, achieving a five-year survival rate exceeding 70% in patients without metastasis (Lu et al. 2022). However, OS has a strong tendency to metastasis, which poses a great challenge for effective treatment (Chen et al. 2022). The outcomes of metastatic OS remains poor, and the 5-year survival rate is < 20% (Lu et al. 2022). Given the bottleneck encountered in the treatment of OS, particularly for patients with metastasis and chemotherapy resistance, the development of novel drugs with high efficiency and safety is still needed.

Plant-derived bioactive compounds are a series of active substances, which exert multiple pharmacological activities in humans, such as anti-inflammation, antioxidant, anti-stress, antiviral, and anti-tumor (Chen et al. 2022). Soy isoflavones (SI) is an active ingredient found in soybeans and is beneficial for the health of the gastrointestinal tract, heart, and brain, among other systems (Al-Nakkash and Kubinski 2020; Sekikawa et al. 2019). As a phytoestrogen, SI is also closely associated with a low incidence of hormone-related cancers such as breast and prostate cancers (Varinska et al. 2015; Sivonova et al. 2019). A meta-analysis based on 8 articles reported that the consumption of SI is associated with a reduced risk of breast cancer in women before and after menopause (Boutas et al. 2022). Another meta-analysis based on 30 articles revealed that the consumption of SI is positively associated with a low risk of prostate cancer (Applegate et al. 2018). In addition, evidence has also determined that SI can sensitize cancer cells to chemoradiotherapy but not affect normal cells (Sahin et al. 2019). However, the detailed effects of SI in OS and relevant molecular mechanisms are rarely reported yet.

Mitophagy, a specialized form of autophagy targeting mitochondria, plays an essential role in maintaining mitochondrial function (Zhang et al. 2022). Mitophagy exerts a crucial role in tumorigenesis and progression, but its function is still controversial in different types of cancers (Panigrahi et al. 2020). As reported, the activation of mitophagy can promote the progression of breast cancer (Li et al. 2022) and hepatocellular carcinoma (Yao et al. 2022). On the contrary, mitophagy exerts a tumor-suppressor role in glioma (Huang et al. 2021), cervical (Sun et al. 2022), ovarian (Meng et al. 2022), colon (Yin et al. 2021), and lung cancers (Hwang et al. 2022). Previous studies have indicated that various potential therapeutic agents, including protodioscin (Huang et al. 2023), ZnO nanoparticles (He et al. 2020), parthenolide (Yang et al. 2016), and norcantharidin (Mei et al. 2019), can inhibit OS progression by inducing mitophagy. Of note, SI also participates in the regulation of mitophagy. Li M et al. reported that genistein alleviates the senescence of bone marrow mesenchymal stem cells through inducing mitophagy (Li et al. 2023). Li et al. revealed that SI protects neurons from the toxicity of atrazine by inducing mitophagy (Li et al. 2021). However, whether the role of SI in OS is associated with mitophagy is still unclear.

The pathogenesis of cancers is complex and involves a multitude of signaling pathways. The AKT/mTOR signaling pathway is regarded as a promising therapeutic target for human cancers (Yu et al. 2022). Evidence has determined that the AKT/mTOR pathway also participates in the process of mitophagy (Zhao et al. 2022; Zheng et al. 2021; Liu et al. 2021). Of note, Zhang et al. reported that SI alleviates hypoxic damage in neuronal cells by blocking the AKT/mTOR pathway (Zhang et al. 2022). Therefore, mitophagy that is mediated by the AKT/mTOR pathway may be involved in the action mechanisms of SI in OS.

In the current research, we examined the effects of SI on OS using both in vitro and in vivo models. Moreover, the action mechanisms of SI involving mitophagy and the AKT/mTOR pathway were analyzed. By delving deeper into these molecular interactions, this study aims to not only shed light on the multifaceted action of SI in OS treatment but also to explore its potential as a novel, efficacious therapeutic agent, thereby contributing to the advancement of cancer therapy.

## Methods

### Cell culture and treatments

Human OS cell lines U2OS, Saos2, and MG63 (Pricella, Wuhan, China; Catalog No: CL-0236, CL-0202, and CL-0157, respectively) were cultured in RPMI-1640 medium (HyClone, Logan, UT, USA; Catalog No: 8120348) with 10% fetal bovine serum (FBS; Ephraim, Xiamen, China; Catalog No: 34080619) at 37 °C with 5% CO_2_. For treatments, U2OS cells were incubated with SI, which was obtained as an analytical standard with 80% purity from Shanghai Yuanye Bio-Technology Co., Ltd. (Shanghai, China; Catalog No: 574-12-9; 10, 20, and 40 μM) for 48 h. In addition, a part of the U2OS cells were pre-treated with 50 mM Mdivi-1 (an inhibitor of mitophagy; Beyotime, Beijing, China; Catalog No: SC8028-5 mg) for 1 h and then incubated with 40 μM SI for another 48 h. Another part of U2OS cells were incubated with 40 μM SI combined with 5 μM SC-79 (an AKT agonist; Beyotime; Catalog No: SF2730-5 mg) for 48 h or pre-treated with 10 nM MK-2206 (an AKT inhibitor; Beyotime; Catalog No: SF2712-10 mM) for 1 h and then incubated with 40 μM SI for another 48 h.

### Cell Counting Kit-8 (CCK-8) assay

Cell viability was assessed using a CCK-8 kit (Beyotime; Catalog No: C0037). Briefly, 100 µL U2OS cells that were seeded in 96-well plates were incubated with SI for 12, 24, 48, and 96 h, respectively. After 2 h of incubation with 10 µL CCK-8, optical density at 450 nm was measured in each well by a microplate reader (DR-3518G, Hiwell Diatek, Wuxi, China).

### Colony-formation assay

Colony-formation assay was performed to reflect the state of cell proliferation. In brief, U2OS cells were seeded into 6-well plates in an initial quantity of 200 cells/well. After 14 days of culturing, the former colonies were stained with crystal violet (Beyotime; Catalog No: C0121-100 mL) for 20 min and counted in each well.

### Wound-healing assay

Wound-healing assay was performed to evaluate cell migration. In brief, U2OS cells were cultured overnight in 6-well plates. A wound track was made in each well along the diameter. After 24 h of continuous culturing, the width of the wound gap was quantified. The migration rate was calculated as (distance^0 h^- distance^24 h^)/distance^0 h^ × 100%.

### Trans-well assay

Trans-well assay was performed to evaluate cell invasion. Briefly, a matrigel-coated upper chamber was added with 200 µL U2OS cells, and the lower chamber was added with RPMI 1640 medium containing 20% FBS. After 24 h, cells in the lower chamber were collected and then stained with crystal violet for 20 min. Invasive cells, stained violet, were quantified under a microscope (CKX53, Olympus, Japan).

### Transmission electron microscopy (TEM)

Mitochondrial autophagosomes were observed by TEM. In brief, U2OS cells were fixed in 2.5% glutaraldehyde (Sinopharm, Shanghai, China; Catalog No: 30092436), dehydrated in alcohol (Sinopharm; Catalog No: 10009218) and acetone (Sinopharm; Catalog No: 10000418), embedded in epoxy resin (Solarbio, Beijing, China; Catalog No: G8590), and sliced. After stained with 2% sodium acetate-lead citrate (Sinopharm; Catalog No: 6131-90-4 and Macklin, Shanghai, China; Catalog No: 512-26-5, respectively), the samples were captured under a TEM microscope (JEM-1400FLASH, JEOL, Japan).

### Immunofluorescence (IF)

The location and expression of LC3 in U2OS cells were detected by IF. Briefly, U2OS cells that were seeded in 12-well plates were stained with Mito-Tracker Green solution (Beyotime; Catalog No: C1048) for 30 min. After being washed with phosphate-buffered saline (Beyotime; Catalog No: C0221A), cells were fixed in 3% methanol (Sinopharm; Catalog No: 80080418), treated with 1% Triton-X 100 (Solarbio, Catalog No: T8200), and blocked with 3% bovine serum albumin (Sigma-Aldrich, St Louis, MO, USA; Catalog No: A7906). Subsequently, cells underwent 12 h of incubation with anti-LC3 (1:250, Abcam, Cambridge, UK; Catalog No: ab192890) at 4 °C, and further underwent 1 h of incubation with Alexa Fluor® 647-IgG (1: 500, Abcam; Catalog No: ab150079) and 4′, 6-diamidino-2-phenylindole (DAPI) (Beyotime; Catalog No: C1005) at 25 ℃. After quenching and sealing, cells displaying fluorescence were observed under a microscope (CKX53, Olympus).

### Flow cytometry

Using an Apoptosis Detection Kit (Beyotime; Catalog No: C1062S), sample cells were labeled with 5 µL Annexin V-FITC and 10 µL propidium iodide to detect apoptosis. Using a Mitochondrial Membrane Potential Detection Kit (Beyotime; Catalog No: C2006), sample cells were labeled with JC-1 to detect mitochondrial membrane potential. Using ROS Detection Kit (Beyotime; Catalog No: S0033S), sample cells were labeled with DCFH-DA fluorescence probe to detect ROS level. Data acquired from the flow cytometer (CytoFLEX S, Beckman, Miami, FL, USA) were analyzed using Cell Quest software (BD Biosciences, Franklin Lake, NJ, USA).

### Oxygen consumption rate (OCR) assay

OCR was detected by an extracellular flux analyzer (XFe96, Agilent, Santa Clara, CA, USA). In brief, U2OS cells that were seeded in XF96e plates were cultured overnight at 37 °C. Subsequently, cells were incubated for 70 min in a CO_2_-free assay medium. During culturing, oligomycin (3 μM; Sigma-Aldrich; Catalog No: 75351), FCCP (0.5 μM; Sigma-Aldrich, Catalog No: C2920), and rotenone combined with antimycin A (both 1 μM; Sigma-Aldrich, Catalog No: R8875 and A8674, respectively) were added at the 20th, 35th, and 50th min, respectively.

### Establishment of tumor xenograft model in mice

BALB/c nude mice (male, 4 weeks old) were obtained from HFK Bioscience (Beijing, China). After a one-week acclimatization period, 100 μL U2OS cells (2 × 10^6^ cells/mL) were subcutaneously injected into the right flank to induce the xenograft OS model. For the construction of a lung metastatic model, 4-week-old male BALB/c nude mice were injected with 100 μL U2OS cells (2 × 10^6^ cells/mL) via the tail vein. After the tumor reached 100 mm^3^, 40 mg/kg SI was gavaged in mice once a day (N = 5). Mice in the control group were gavaged with an equal volume of physiological saline (N = 5). Via measuring tumor diameter every 4 days, the tumor volume was calculated as: 0.5 × (diameter_longest_ × diameter_shortest_^2^). Twenty-eight days later, mice were anesthetized with 50 mg/kg pentobarbital sodium and then sacrificed by cervical dislocation. The tumor xenografts were resected, photographed, and weighted. All animal experimental procedures were sanctioned by the Ethics Committee of Yangzhou University School of Medicine and were conducted following the Guide for the Care and Use of Laboratory Animals (202303137).

### TUNEL staining and immunohistochemistry (IHC)

TUNEL staining and IHC were performed to detect the apoptosis and proliferation of tumor cells in vivo, respectively. In brief, the xenograft samples were fixed in 4% paraformaldehyde (Sinopharm; Catalog No: 10010018), dehydrated in graded ethanol, vitrificated in xylene (Sinopharm; Catalog No: 10023418), embedded in paraffin, and sliced at 5–7 μm. For TUNEL staining, the sliced samples were stained with TUNEL (Beyotime; Catalog No: C1086) for 60 min and then with DAPI for another 10 min under darkness. For IHC, the sliced samples were incubated with anti-Ki67 (1:200, Abcam; Catalog No: ab15580) for 12 h, and then with horseradish peroxidase (HRP)-conjugated IgG (1:500, Abcam; Catalog No: ab205718) for another 1 h. After incubation with diaminobenzidine (Beyotime; Catalog No: p0203) for 30 min under darkness, the samples were re-stained with hematoxylin for another 3 min. After quenching and sealing, the stained sections were observed under a microscope (CKX53, Olympus).

### Hematoxylin and eosin (HE) staining

Lung tissue samples were fixed in 4% paraformaldehyde for 24 h, embedded in paraffin wax, and cut into 4–7 μm sections. The sections were immersed in xylene for 15 min, followed by rehydration through a graded alcohol series. Then, sections were stained with hematoxylin solution for 5 min and eosin solution for 1 min. After being mounted with a resinous, the sections were observed under a microscope to assess the histological features.

### Western blot

Total proteins in U2OS cells or tumor xenografts were extracted by lysing in RIPA buffer (Beyotime; Catalog No: P0013B). Equal amounts of protein from different groups were separated via 8% sodium dodecyl sulfate–polyacrylamide gel electrophoresis and transferred to polyvinylidene fluoride membranes. Subsequently, the membranes carrying protein samples were blocked with blocking buffer (Beyotime; Catalog No: P0216-300 g) for 1 h, incubated with specific primary antibody for 12 h, and further incubated with specific secondary antibody (HRP-conjugated IgG, 1:2000, Abcam) for 1 h under darkness. After visualizing using a hypersensitive chemiluminescence kit (Beyotime; Catalog No: P0018S), the blots were analyzed by a Gel Imaging System (3500, Tanon, China). The primary anti-bodies used in western blot included anti-Bax (1:5000, Abcam; Catalog No: ab32503), -Bcl-2 (1:1000, Abcam; Catalog No: ab32124), -pro caspase-3 (1:1000, Abcam; Catalog No: ab32150), -cleaved caspase-3 (1:5000, Abcam; Catalog No: ab214430), -P62 (1:1000, Abcam; Catalog No: ab207305), -LC3 (1:2000, Abcam; Catalog No: ab192890), -PINK1 (1:1000, Abcam; Catalog No: ab216144), -Parkin (1:1000, Cell Signaling Technology, Danvers, MA, USA; Catalog No: 2132S), -AKT (1:5000, Abcam; Catalog No: ab227385), -p-AKT (phospho T308; 1:5000, Abcam; Catalog No: ab38449), -mTOR (1:5000, Abcam; Catalog No: ab134903), p-mTOR (phospho S2448; 1:5000, Abcam; Catalog No: ab109268), and -GAPDH (1:5000, Abcam; Catalog No: ab181602).

### Statistical analysis

Measurement data were expressed as mean ± standard deviation and were statistically analyzed by GraphPad Prism 7.0 (San Diego, CA, USA). Two-group comparisons were made using an independent t-test, while multiple-group comparisons were made using one-way ANOVA followed by Tukey's post hoc test. A P value < 0.05 represented a statistically significant.

## Results

### SI suppresses the malignant features of OS cells

**Fig. 1:**
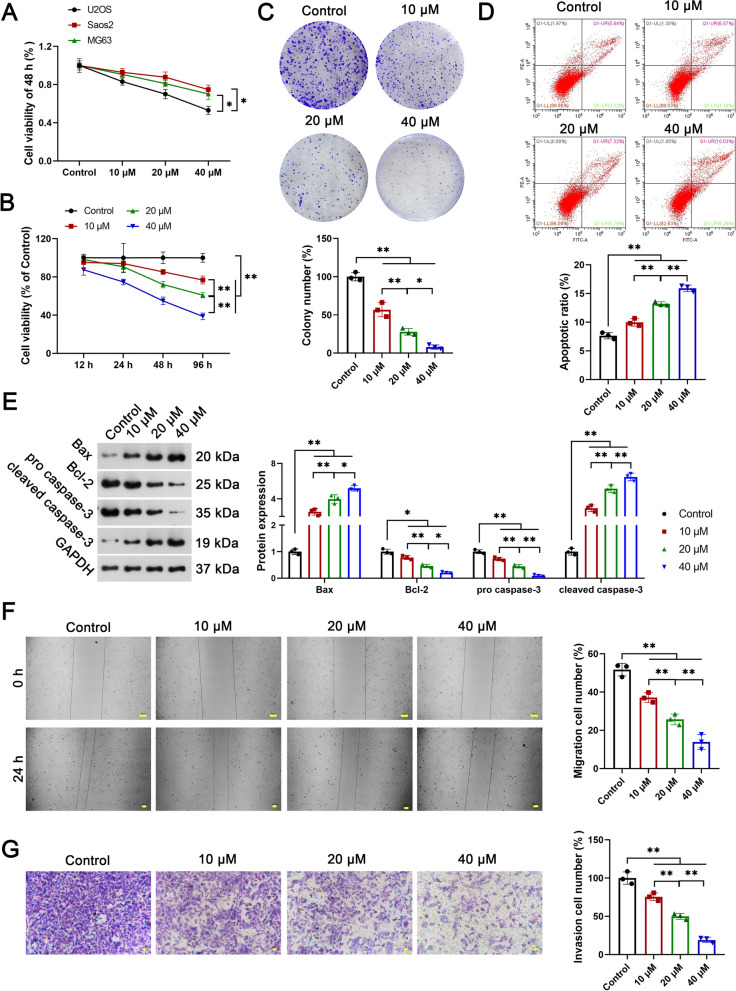
SI inhibits the malignant characteristics of OS cells. **A**, **B** Cell viability was measured by CCK-8 assay; **C** colony number was measured by colony formation assay; **D** cell apoptosis was measured by flow cytometry; **E** the protein expression of Bax, Bcl-2, Pro caspase-3, and Cleaved caspases-3 were measured by Western blot; **F** cell migration was measured by wound-healing assay; G, Cell invasion was measured by trans-well assay. Human OS cell lines U2OS, Saos2, and MG63 were treated with 10 μM, 20 μM, or 40 μM SI for 48 h. All experiments were repeated three times; statistical analysis was performed using one-way ANOVA followed by Tukey's post hoc test. ^*^P < 0.05, ^**^P < 0.01

Initially, the therapeutic potentials of SI (10, 20, and 40 μM) were assessed against a panel of OS cell lines, namely U2OS, Saos2, and MG63. Notably, U2OS cells exhibited heightened sensitivity to SI, showcasing superior inhibition in cell viability as compared to Saos2 and MG63 cells (P < 0.05, Fig. 1A). Consequently, U2OS was selected as the cell line of choice for all subsequent experimental investigations. The viability and colony number of U2OS cells were significantly reduced in a dose-dependent manner following SI treatment (P < 0.05, Fig. 1B and C). SI also enhanced the apoptosis of U2OS cells in a dose-dependent manner, as evidenced by a decrease in the apoptotic ratio (P < 0.01, Fig. 1D), up-regulation of Bax and Cleaved caspases-3, and down-regulation of Bcl-2 and Pro-caspase-3 (P < 0.05, Fig. 1E). Furthermore, significantly fewer cells for migration and invasion were observed in the SI group compared with the control group (P < 0.01). Cell migration and invasion were similarly attenuated with increasing concentrations of SI in U2OS cells (P < 0.01, Fig. 1F and G).

### SI induces mitophagy in OS cells

**Fig. 2:**
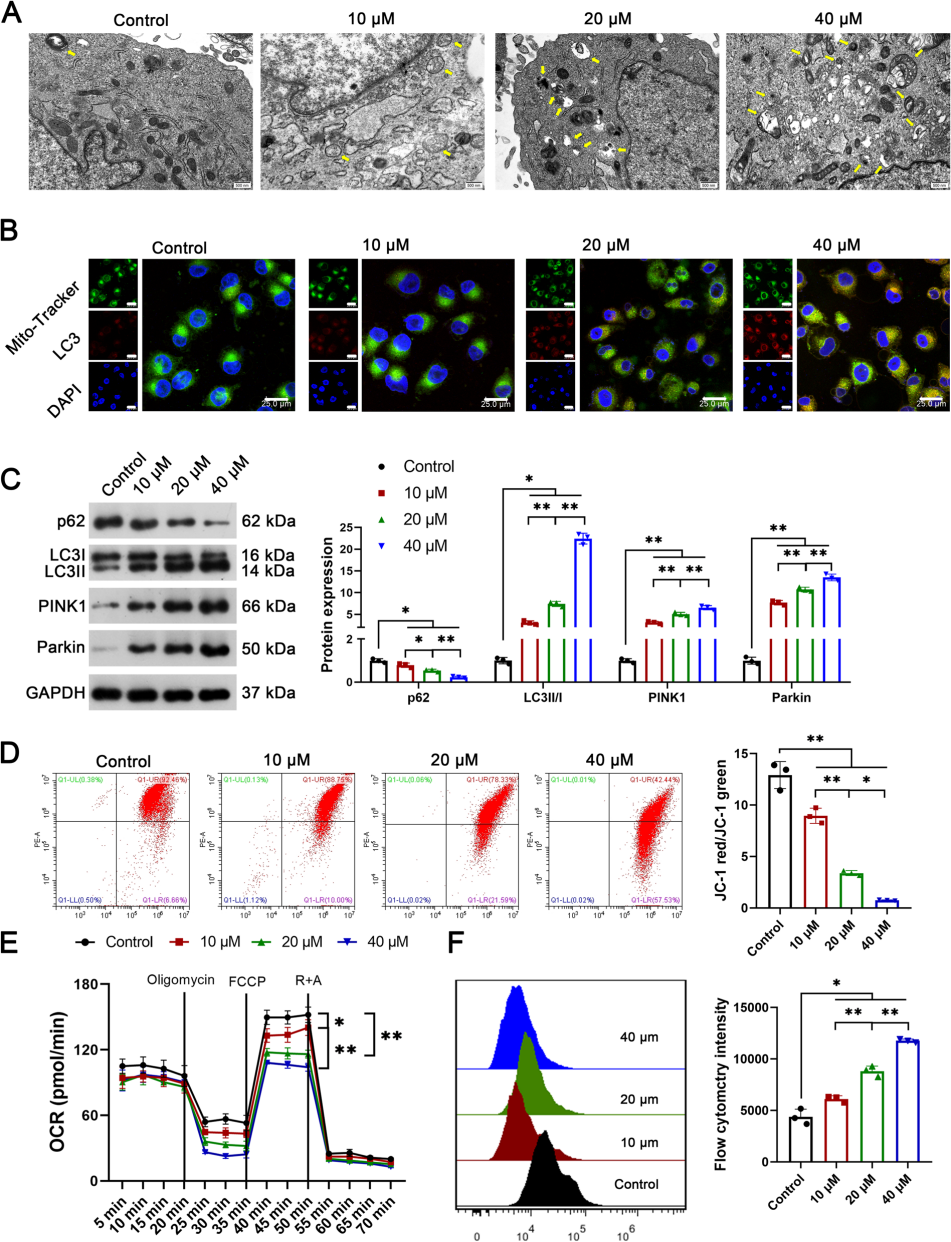
SI induces mitophagy in OS cells. **A** Mitochondrial autophagosomes were observed under TEM; **B** the location and expression of LC3 were detected by IF; **C** the protein expression of P62, LC3 II/I, PINK1, and parkin (autophagy-related proteins) were detected by western blot; **D** the mitochondrial membrane potential (JC-1 red/green) was detected by flow cytometry; **E** the OCR was measured by an extracellular flux analyzer; **F** the ROS was measured by flow cytometry. Human OS cell lines U2OS, Saos2, and MG63 were treated with 10 μM, 20 μM, or 40 μM SI for 48 h. All experiments were repeated three times; statistical analysis was performed using one-way ANOVA followed by Tukey's post hoc test. ^*^P < 0.05, ^**^P < 0.01

The effects of SI on mitophagy were subsequently explored in U2OS cells. Under TEM, a higher number of mitochondrial autophagosomes were observed in SI-treated U2OS cells than in the controls, and the number of autophagosomes increased with increasing concentrations of SI (Fig. 2A). Under a fluorescence microscope, LC3 protein that co-located with mitochondria was dose-dependently up-regulated by the intervention of SI (Fig. 2B). SI also dose-dependently down-regulated P62, but up-regulated LC3 II/I, PINK1, and parkin (autophagy-related proteins) in U2OS cells (P < 0.05, Fig. 2C). Additionally, the mitochondrial membrane potential was weakened in a dose-dependent manner following SI treatment, as evidenced by a significant decrease in JC-1 red/green (P < 0.05, Fig. 2D). The OCR was similarly decreased in a dose-dependent manner following SI treatment (P < 0.05, Fig. 2E). Moreover, a dose-dependent increase in ROS was observed in SI-treated U2OS cells compared to the controls (P < 0.05, Fig. 2F).

### The anti-tumor potential of SI in OS is related to mitophagy activation

**Fig. 3:**
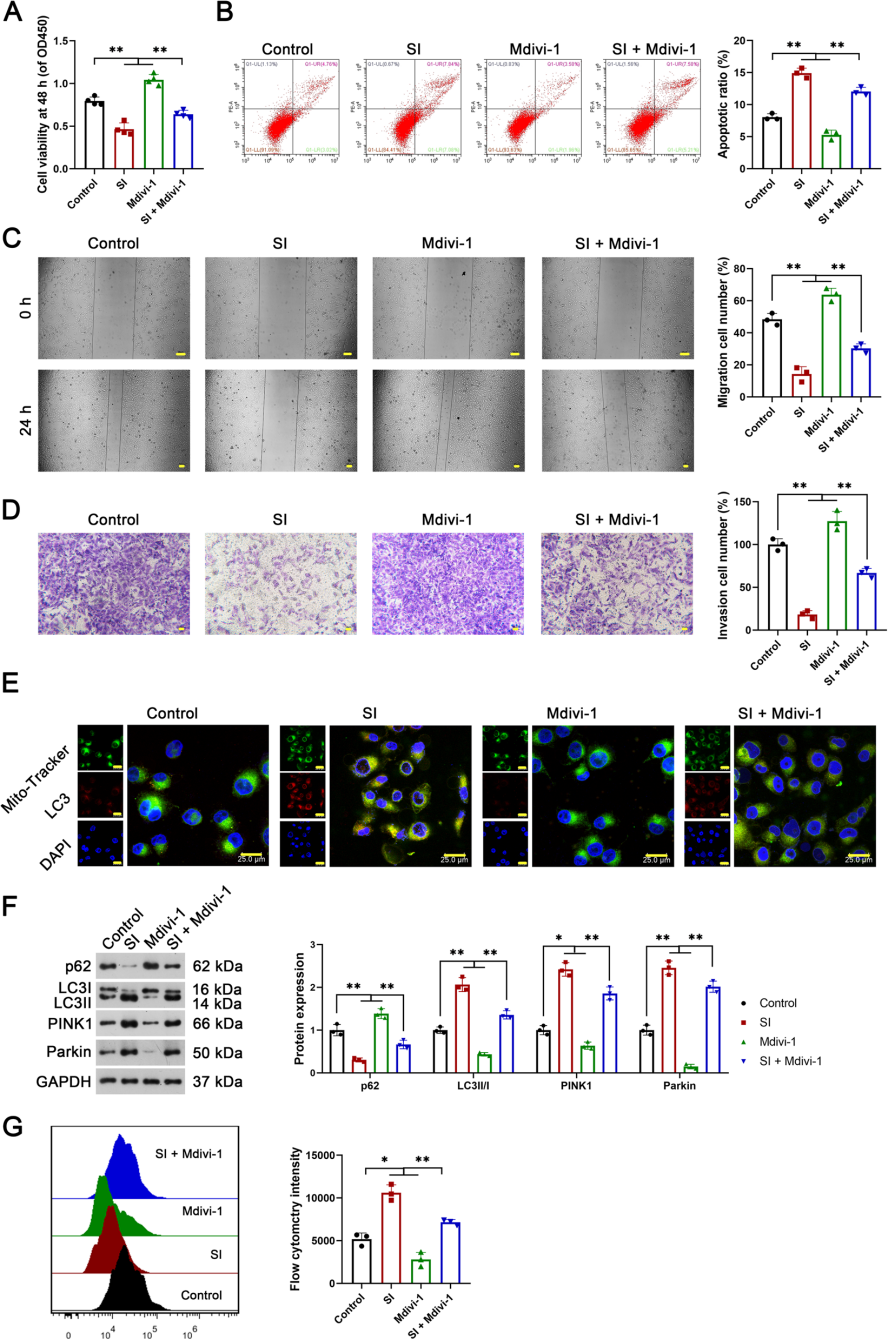
Inhibition of mitophagy reversed the anti-tumor effects of SI on OS cells. **A** Cell viability was measured by CCK-8 assay; **B** cell apoptosis was measured by flow cytometry; **C** cell migration was measured by wound-healing assay; **D** cell invasion was measured by trans-well assay; **E** the location and expression of LC3 were detected by IF; **F** the protein expression of P62, LC3 II/I, PINK1, and parkin (autophagy-related proteins) were detected by western blot; **G** The ROS was measured by flow cytometry. U2OS cells were pre-treated with 50 mM autophagy inhibitor Mdivi-1 for 1 h and then treated with 40 μM SI for 48 h. All experiments were repeated three times; statistical analysis was performed using one-way ANOVA followed by Tukey's post hoc test. ^*^P < 0.05, ^**^P < 0.01

To reveal the anti-tumor mechanisms of SI about mitophagy activation, Mdivi-1, an inhibitor of mitophagy, was used for intervention in U2OS cells. As shown in Fig. 3A–D, Mdivi-1 enhanced the viability, migration, and invasion, and reduced the apoptosis of U2OS cells to significant levels (P < 0.01). Mdivi-1 also significantly inhibited the mitophagy of U2OS cells, as evidenced by the down-regulation of P62, and up-regulation of LC3 II/I, PINK1, and parkin (P < 0.01, Fig. 3E and F). Importantly, Mdivi-1 reversed the effects of SI on inhibiting malignant features and inducing mitophagy in U2OS cells (P < 0.01, Fig. 3A–F). Moreover, SI-induced elevation of ROS levels in U2OS cells was weakened by Mdivi-1 intervention (P < 0.01, Fig. 3G).

### The role of SI in inducing mitophagy is related to the inhibition of the AKT/mTOR pathway

**Fig. 4:**
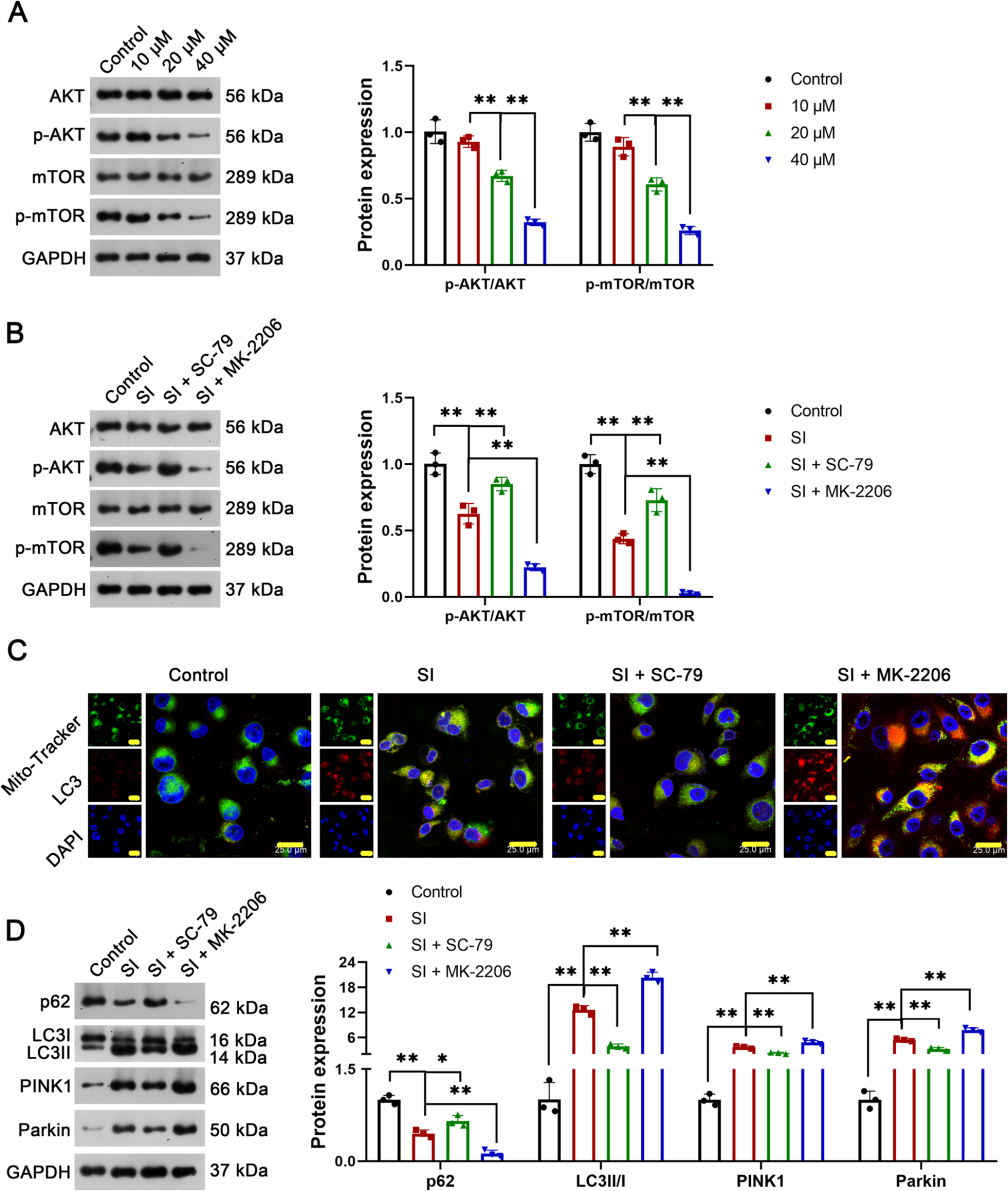
The activation of the AKT/mTOR pathway reversed the effects of SI on inducing mitophagy in OS cells. **A** The protein expression of p-AKT/AKT and p-mTOR/mTOR in U2OS cells treated with SI were detected by western blot; U2OS cells were treated with 10 μM, 20 μM, or 40 μM SI for 48 h. **B** The protein expression of p-AKT/AKT and p-mTOR/mTOR in U2OS cells treated with SI combined with SC-79 (AKT agonist) or MK-2206 (AKT inhibitor) were detected by western blot; **C** the location and expression of LC3 were detected by IF; **D** the protein expression of P62, LC3 II/I, PINK1, and parkin (autophagy-related proteins) were detected by western blot. U2OS cells were co-treated with 40 μM SI and 5 μM SC-79 (AKT agonist) or 10 nM MK-2206 (AKT inhibitor) for 48 h. All experiments were repeated three times; statistical analysis was performed using one-way ANOVA followed by Tukey's post hoc test. ^*^P < 0.05, ^**^P < 0.01

The action mechanisms of SI implicating the AKT/mTOR signaling pathway were subsequently explored. As presented in Fig. 4A, SI at 20 and 40 μM significantly down-regulated p-AKT/AKT and p-mTOR/mTOR in U2OS cells (P < 0.01). The effect of SI at 40 μM was stronger than that at 20 μM in inhibiting the AKT/mTOR pathway (P < 0.01). Subsequently, the AKT/mTOR pathway was actively activated using SC-79 (AKT agonist) or blocked using MK-2206 (AKT inhibitor) in U2OS cells (P < 0.01, Fig. 4B). SC-79 reversed the effects of SI on down-regulating P62, and on up-regulating LC3 II/I, PINK1, and parkin (P < 0.05). In contrast, MK-2206 aggravated the effects of SI on inducing mitophagy (P < 0.01, Fig. 4C and D).

### *SI inhibits the growth and metastasis of OS xenografts *in vivo

**Fig. 5:**
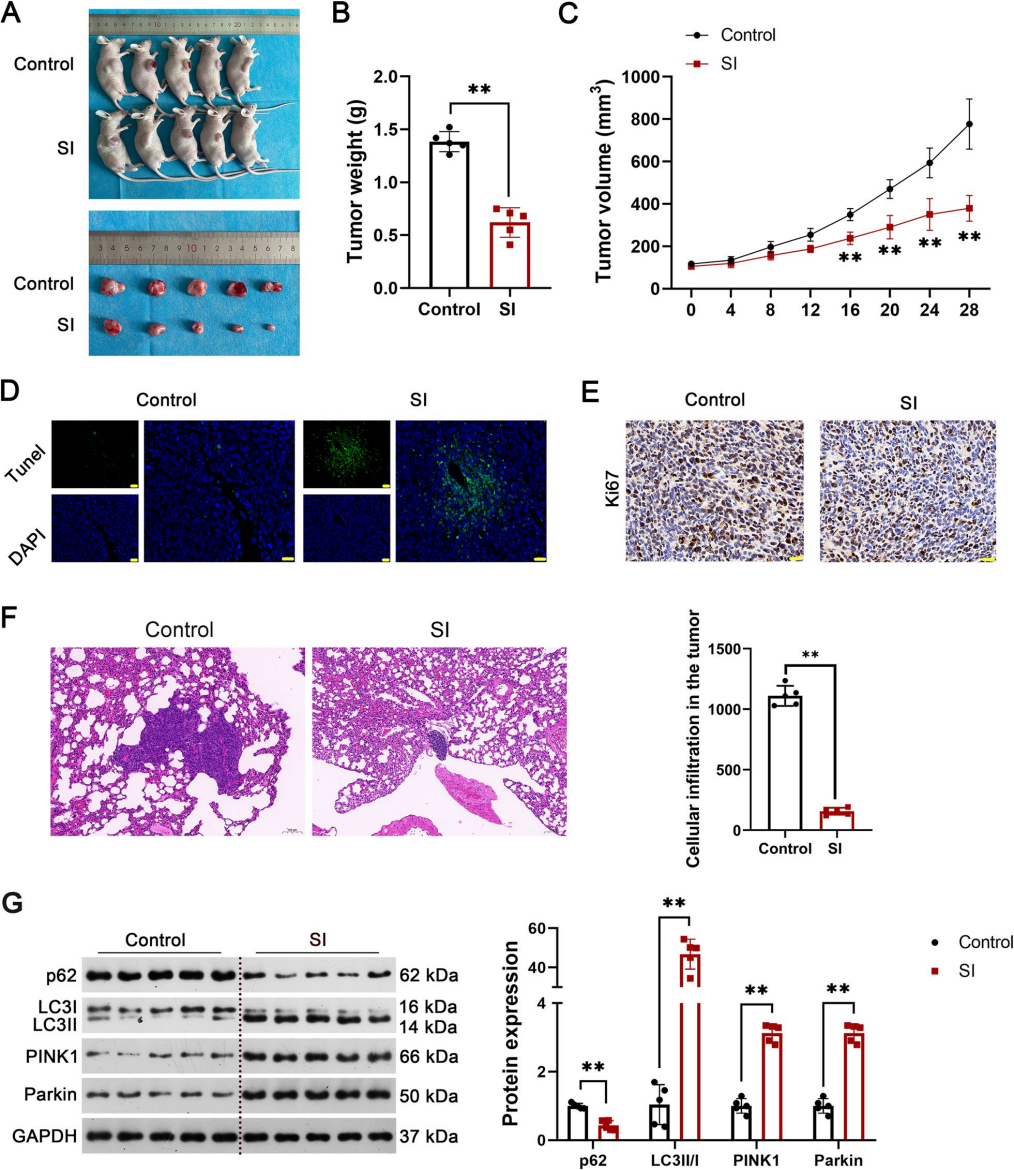
SI inhibits the growth of tumor xenografts in mice. **A** The morphology of the mouse model and separated tumor xenografts; **B** the tumor weight on the 28th day; **C** the tumor volumes every 4 days; **D** the apoptotic cells were detected by TUNEL staining; **E** the active cells were detected by IHC of Ki67; **F** HE staining for lung tissues; **G** the protein expression of P62, LC3 II/I, PINK1, and parkin (autophagy-related proteins) were detected by western blot. OS model mice were gavaged with 40 mg/kg SI once a day for 28 days. Experiments were performed with five mice per group; statistical analysis was performed using an independent t-test. ^**^P < 0.01

The effects of SI in OS were further verified in a xenograft mouse model. As presented in Fig. 5A–C, the gavage of SI inhibits the growth of tumor xenografts in mice, as evidenced by the significant decrease in tumor weight on the 28th day and in tumor volume beginning from the 16th day (P < 0.01). In addition, more apoptotic cells positive for TUNEL and less active cells positive for Ki67 were observed in SI-treated mice compared with the controls (Fig. 5D and E). HE staining revealed obvious cellular infiltration in the lung tissues (Fig. 5F). However, SI significantly inhibited the metastasis of OS toward the lung (P < 0.01, Fig. 5F). Moreover, western blot revealed that the gavage of SI significantly down-regulated P62, and up-regulated LC3 II/I, PINK1, and parkin in tumor xenografts (P < 0.01, Fig. 5G).

## Discussion

OS is a prevalent bone malignancy characterized by a high mortality rate, primarily due to its strong tendency for pulmonary metastasis (Cui et al. 2020). Despite advancements in therapeutic regimens, the clinical outcomes for patients with OS remain unsatisfactory. In the present study, the anti-tumor effects and mechanisms of SI on OS were explored. Our study revealed that SI suppressed the malignant characteristics of OS cells in vitro, as well as the growth of OS xenografts in vivo. The activation of mitophagy, mediated by the AKT/mTOR pathway, is involved in the action mechanisms of SI in OS.

Plant-derived bioactive compounds are active substances that received much attention in clinical use. More and more studies have revealed that plant-derived bioactive compounds exhibit promising therapeutic potential against tumors, such as glycosides, flavonoids, polyphenols, polysaccharides, phenols, quercetin, etc. (Esmeeta et al. 2022; Raju et al. 2022). SI belongs to the class of phytoestrogens and possesses a structural similarity to the human 17-beta estradiol hormone (Boutas et al. 2022). SI exerts beneficial effects on lowering blood lipids and glucose, inhibiting oxidative stress, preventing osteoporosis, and reducing the risk of breast and prostate cancer (Varinska et al. 2015; Sivonova et al. 2019; Nakai et al. 2020). Genistein and daidzein are two major active constituents of SI, exhibiting significant biological functions in humans (Song et al. 2015). By affecting cell apoptosis, cell cycle, metastasis, and angiogenesis, genistein exhibits great therapeutic potential against diverse types of cancers (Spagnuolo et al. 2015). Similarly, daidzein also exerts anti-tumor activity against lung cancer (Guo et al. 2020), breast cancer (Alshehri et al. 2021), bladder cancer (He et al. 2016), ovarian cancer (Hua et al. 2018), etc. Of note, Song et al. demonstrated that genistein suppresses the viability and enhances the apoptosis of OS cells (Song et al. 2015). Zhu et al. revealed that daidzein suppresses the proliferation and migration of OS cells (Zhu et al. 2021). Here, the effects of SI on the malignant features of OS were explored. As a result, SI dose-dependently suppressed the proliferation, migration, and invasion, and enhanced the apoptosis of U2OS cells. These results are similar to previous studies on genistein and daidzein mentioned above and demonstrate a positive role of SI in inhibiting OS cells in vitro. Furthermore, our in vivo experiments corroborated these findings, demonstrating that SI not only reduced tumor volume and weight but also increased the proportion of apoptotic cells while decreasing the number of active cells in OS xenografts. Our findings confirmed a promising efficiency of SI in the treatment of OS.

Mitophagy is a conserved intracellular process that is important for mitochondrial health. Via removing dysfunctional or superfluous mitochondria in cells, mitophagy plays a crucial role in various human diseases, including cardiovascular, neurodegenerative, and metabolic diseases, and cancers (Lu et al. 2023). In this study, the mitophagy was activated in OS cells by the intervention of SI, evidenced by the increase in autophagosomes and ROS, a decrease in mitochondrial membrane potential and OCR, and changes in autophagy-related proteins. As previously reported, the activation of mitophagy contributes to the treatment of cervical cancer (Sun et al. 2022), colon cancer (Yin et al. 2021), glioma (Huang et al. 2021), ovarian cancer (Meng et al. 2022), lung cancer (Hwang et al. 2022), etc. The anti-tumor role of some agents in OS is also related to the activation of mitophagy. For example, protodioscin inhibits the viability and enhances the apoptosis and mitophagy of OS cells (Huang et al. xxxx). The effects of norcantharidin on inhibiting cell proliferation, migration, and invasion are related to mitophagy in OS (Mei et al. 2019). Parthenolide activates mitophagy-mediated cell death in OS (Yang et al. 2016). Based on these findings, our findings suggest that the activation of mitophagy may participate in the anti-tumor mechanisms of SI in OS. To further verify this regulatory mechanism, mitophagy was actively inhibited using Mdivi-1 in subsequent experiments. Encouragingly, mdivi-1 reversed the effects of SI on inhibiting the malignant characteristics of U2OS cells. Therefore, SI may inhibit the progression of OS by inducing mitophagy.

Many signaling pathways participate in the initiation and progression of OS, such as the Wnt/β-catenin, AKT/mTOR, Notch, HIF-1α, P53, MAPK, and JNK pathways (Han and Shen 2020). Among them, the AKT/mTOR pathway is a well-known regulator in multiple cellular functions (Alzahrani 2019). In this study, the AKT/mTOR pathway was significantly inhibited in OS cells following SI treatment, a finding that is consistent with a previous study demonstrating that SI alleviates hypoxic damage in neuronal cells via blocking the AKT/mTOR pathway (Zhang et al. 2022). In addition, the AKT/mTOR pathway also plays a key regulatory role in mitophagy. Zhao et al. reported that leonurine activates mitophagy to protect against oxidative stress-induced damage in bone mesenchymal stem cells by blocking the PI3K/Akt/mTOR pathway (Zhao et al. 2022). Zheng et al. revealed that rapamycin inhibits apoptosis and enhances the mitophagy of neuronal cells by blocking the PI3K/AKT/mTOR pathway (Zheng et al. 2021). Liu et al. demonstrated that Zhen-Wu-Tang activates mitophagy to relieve renal mitochondrial dysfunction via inhibiting the PI3K/AKT/mTOR pathway (Liu et al. 2021). Combined with the positive effects of SI on inducing mitophagy, we suspect that SI may induce the mitophagy of OS cells by blocking the AKT/mTOR pathway. Encouragingly, our following experiments verified this speculation, as evidenced by that the activation of the AKT/mTOR pathway reversed the effects of SI on inducing mitophagy.

## Conclusions

In conclusion, our findings demonstrate that SI exerts anti-tumor effects in OS both in vitro and in vivo, positioning it as a potentially promising therapeutic agent. The therapeutic potential of SI in OS appears to be intricately linked with the activation of mitophagy, which is mediated via the AKT/mTOR signaling pathway.

## Data Availability

The datasets used and analysed during the current study are available from the corresponding author on reasonable request.

## Abbreviations

SI: Soy isoflavones
OCR: Oxygen consumption rate
IHC: Immunohistochemistry
OS: Osteosarcoma
CCK-8: Cell Counting Kit-8
TEM: Transmission electron microscopy
IF: Immunofluorescence
HRP: Horseradish peroxidase
